# An Informational Algorithm as the Basis for Perception-Action Control of the Instantaneous Axes of the Knee

**DOI:** 10.4172/2165-7025.1000127

**Published:** 2013-03-27

**Authors:** Wangdo Kim, Margarida M. Espanha, António P. Veloso, Duarte Araújo, Filipa João, Luis Carrão, Sean S. Kohles

**Affiliations:** 1 Univ Tecn Lisboa, Fac Motricidade Humana, CIPER, LBMF, SPERTLAB, Estrada da Costa, P-1499-002 Lisbon, Portugal; 2 Regenerative Bioengineering Laboratory, Departments of Mechanical and Materials Engineering and Biology, Portland State University, Portland, Oregon, USA

**Keywords:** Bellman’s principle of optimality, Ball-Disteli diagram, Perception-action coupling manifold, Gibson’s theory of affordance, Ball’s screw theory, minimum information principle, Muscle synergies

## Abstract

Traditional locomotion studies emphasize an optimization of the desired movement trajectories while ignoring sensory feedback. We propose an information based theory that locomotion is neither triggered nor commanded but controlled. The basis for this control is the information derived from perceiving oneself in the world. Control therefore lies in the human-environment system. In order to test this hypothesis, we derived a mathematical foundation characterizing the energy that is required to perform a rotational twist, with small amplitude, of the instantaneous axes of the knee (IAK). We have found that the joint’s perception of the ground reaction force may be replaced by the co-perception of muscle activation with appropriate intensities. This approach generated an accurate comparison with known joint forces and appears appropriate in so far as predicting the effect on the knee when it is free to twist about the IAK.

## Introduction

**Figure 1:**
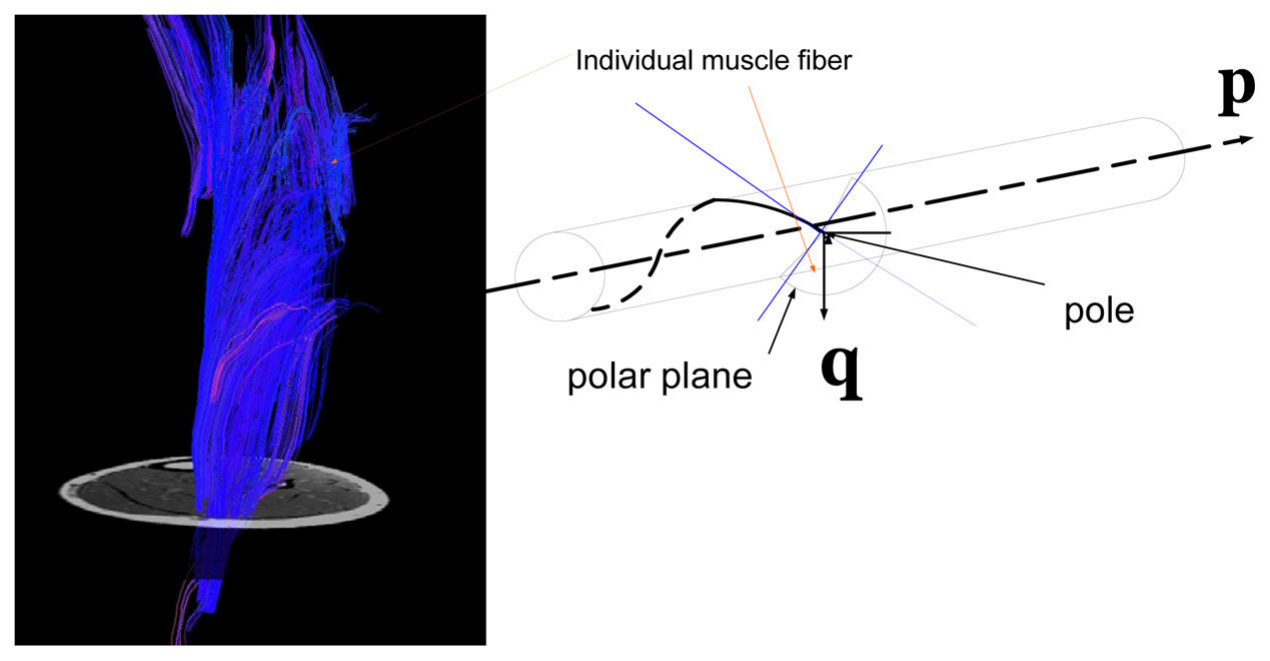
The fiber tractographic results of a portion of the gastrocnemius lateralis muscle generated in a healthy subject. The images were generated with one region of interest, correspondent to the muscle boundary where the anatomical cross-section area was maximal. The subject was examined in a supine position within a 1.5 Tesla whole-body MRI scanner (Signa HD×T 1.5T, GE Healthcare, USA), with the long axis of the leg placed parallel to the magnetic field. A manifold of muscle fibers, when contracted, represents the linear complex defined by a single twist *IAK*(**p**). Every point along the fiber segment has its velocity vector tangential to the helix that passes through it. The overall pattern of this velocity vector is a helicoidal velocity field. Each point that does not coincide with the twist axis *IAK*(**p**) is referred to as a ‘pole.’ Associated with each pole is its corresponding polar plane. As shown here, a polar plane and its corresponding pole are defined by the instantaneous screw axis *IAK*(**p**).

Bernstein [1] recognized that effectors (feed-forward sensors) were not the only important components to movement; feedback was also necessary. It is clear that human locomotion may be studied from a number of different points of views (e.g., anatomical, biological, mechanical). Our interest here is in the control of skeletal activities, specifically, the stance phase of gait; when the leg is nearly fully extended and the foot is in contact with a reaction surface. Neptune used the optimization algorithm fine-tuned the muscle excitation patterns for each muscle group to produce a well-coordinated walking pattern that emulated the experimental data [2–4]; however, in order to reduce the number of musculoskeletal degrees of freedom (DOFs) upon which the nervous system must operate, we have adopted the proposition that the nervous system controls muscle synergies, or groups of co-activated muscles, rather than individual muscles [5,6]. The muscle synergy is equivalent to the complex of lines, a manifold arrangement approximated by individual fibers (Figure 1).

Unique features of the muscular control system are not only in the biological nature of its actuators, but also the specific ways in which the control information is processed [7]. The sensorimotor information is transformed into control signals passing through neural networks. Traditional emphasis has been on optimizing desired movement trajectories while ignoring sensory feedback. Recent work [6] has redefined optimality in terms of feedback control laws, and focused on the mechanisms that generate behavior online. In the case of skeletal control, the internal mechanism describing ways in which the input information is transformed into the output for muscular activation, is lacking [8]. Understanding the complex interplay between neural circuits and biomechanics that give rise to muscle synergies will be crucial to advancing our understanding of neural control mechanisms for movement [5].

So far, two approaches in studying of the neuromuscular system have been used. Advances in electroencephalography have made it possible to study the correlation of different levels of neural system and muscular activity [9]. At the other extreme, the peripheral neuromuscular blocks, meaning the local organization of neurons, muscles, skeletal elements, and associated sense organs, has also been made the subject of several studies [10,11]. From a control engineering point of view, the models of peripheral block are derived basically by using the theory of servo mechanisms.

The problem, however, of coordination of the different muscle groups involved in the repetitive sequence of skeletal activities, such as locomotion [12], has been given limited attention from the point of view of control theory when random effects [13] can arise in the control process. In fact, the skeleton with all its actuators represents a typical large control system. Therefore it is interesting to explore what result can be obtained by using a systematic approach to muscle control. In this article, we present the concept that control lies in the human-environment system which fits into an existing literature of ‘ecological psychology’.

Human movement control can be seen as a process that is distributed over the performer-environment system, i.e., rather than being localized in an internal structure within the performer [14]. A recent study [15] confirmed that leg stiffness is not directly related to running mechanics, but rather, to the running environment. Other studies also found that the stiffness of the leg might be altered by changing the activation of muscles acting about the joints of the leg [16,17]. The relationship between stiffness (vertical, leg and joint parameters) and running surfaces was studied using spring-mass system models [18–21].

The adjustability of leg stiffness may be important in allowing the body’s spring system to operate over the variety of terrain encountered in the natural world [22]. The performer and his/her environment (surface) may be said to be co-participants in any resulting action. In this way, actions are specific to function rather than to mechanism [23]. Movements and postures are controlled and coordinated to realize functionally specific acts that are themselves based on the perception of affordances, i.e., possibilities for actions [14]. Therefore, during our study of locomotion, we first investigated the complementarities between the perception of the surface in terms of the ground reaction forces (GRF) and the co-perception of the self in terms of the instantaneous axes of the knee (IAK), as the two are inseparable [24,25]. We have enunciated a principle, which applies to the reciprocal screw system and involves the theory of equilibrium that has freedom of the first order.

The term haptic perception refers to the perception that is itself based on feedback from the mechano-receptive machinery that is embedded in the body’s deformable tissues. Haptic perception is perception by means of the body, in concert with the general definition of perceptual systems [26,14]. It functions in two distinct ways, each of which may act either in isolation or concurrently: a) proprioceptively–perception of the body and perception of the body’s segments relative to the body as a unit and relative to each other, and; b) exteroceptively–perception of attachments to the body (e.g. handheld objects) and of surfaces adjacent to the body [27]. The haptic system participates both in perception and in action control, as the locomotion operates with combined twist and wrench control in which the resultant wrench of constraint is a screw system reciprocal to the IAK, while the desired twist is a rotation about the same axis.

We propose that muscle synergies may therefore represent the bottom of a hierarchal neural control structure in which higher neural centers operate on increasingly conceptual variables related to task-level motor performance [5]. In addition some new minimum information concepts will be introduced that seem more appropriate for skeletal activity and that appear to have some significance for the general theory of perception-to-action control. Therefore, we present a technique of directly generating the IAK during the stance phase that is associated with reciprocal connections, based on the minimum information principle. We chose the IAK and GRF and then used their instantaneous successive correspondence during the stance phase to generate two information surfaces. These were then used to create the perception-action coupling manifold.

The secondary aim of our study is to establish the control law based on the reciprocal connection between the IAK and GRF. While our focus was not on the exploration of computational efficiency, some initial results in the medial and lateral compartment forces are presented as a first approximation to validate our method.

## Materials and Methods

### Minimal informational framework

In order to explain a simplified model for locomotion, utilizing minimum information rather than how various muscle groups are involved, we must introduce new learning models that are based on the nature of motor control. The problem, however, is how to coordinate the various muscle groups involved and the respective sequence of skeletal participation, as it occurs during locomotion. Also, we need to consider the environmental circumstances, i.e., the surfaces, where the movement takes place. In fact, the model used in this study represents a typical movement system when the object, i.e., the foot, is grounded. To begin to answer these puzzling questions, we applied some essential knowledge of the spatial gearing that elucidates the relative motion between a pair of bodies.

The way that functional movement of a joint is constrained is related both to the location and the direction of its axes. In other words, the kinematic constraints of joints are line-dependent. This in turn implies that the constraints may be expressed in terms of line- rather than point-based geometry as in screw theory (Ball, 1998). Screw theory is based on the close relationship between line geometry and spatial kinematics [28]. It has previously been used to characterize knee function [29,30], to explore the effectiveness of the golf swing [31], and as a method of minimizing interference from motion data [31]. Plücker Coordinates [28] were extended here so as to describe screw motion, i.e., the reduction of the displacement of a rigid body to its simplest form, and to locate the IAK in terms of the ISA (Instantaneous Screw Axes). Plücker showed that if a body which has freedom of the first order (one DOF) is in equilibrium, and if any screw motion about a certain axis is defined by the lines forming constraint forces as a complex of the fifth order (5 DOFs), then these lines still remain within the complex and the relationship between the two lines is considered ‘reciprocal’.

The central idea behind a screw system with one DOF was feedback control. The basic injunction of feedback control is that the system should not be time-oriented but event-oriented. Obviously, this way of handling control processes specifically, and decision-making in general, is more flexible than being based solely on voluntary actions. Furthermore, this technique can handle uncertainties easily as it treats the approach as a deterministic process and allows for a special configuration in which the screw system may be applied *transitorily* to match the desired compliance during the stance phase. From the standpoint of decision-making, we are herewith employing the concept of *policy*, whereas the control action that is taken depends on the state of the mechanical system.

### A Screw system with one dof control

In order to illustrate a screw system with a perception-action control scenario, a tractographic reconstruction of the diffusion tensor data derived from the muscle fiber tracking of the gastrocnemius lateralis muscle was performed (Diffusion Toolkit and TrackVis, Department of Radiology, Massachusetts General Hospital, USA). The visualized action manifold contains muscle fibers characterized through approximately 600 tracks (Figure 1). The mean length (± standard deviation) of the tracks (representing the individual fibers) was measured as 66.9 ± 13.66 mm. The image of the gastrocnemius lateralis presented here originally had 3,474 tracks before filtering in order to facilitate the observation of the fiber directions.

For a screw **p** to belong to a screw system of the fifth order (5 DOFs), the necessary and sufficient condition is that **p** be reciprocal to one given screw **q** (Figure 1). This condition is expressed in the form:

(1) $$R(p,q)=(h+h^{\prime })\cos\theta -a\sin\theta =0$$

where *h* and *h*′ are pitches of screw **p** and **q**, respectively, θ is the angle between the two screw axes and **a** is the normal distance between the two axes. The expression defined by equation (1) is referred to as the virtual coefficient of a pair of screws and is of great importance in the present study because it provides an expression for the energy required to affect the displacement or muscle synergies used for equilibrium control during the stance phase (as stated earlier).

To define a one DOF control law of the simplest type, we started with the original notion of a multistage perception process (or Marcov process). We now describe the importance concept of a policy of action. As indicated above, the value of **q***_k_* are to be chosen at each stage in a *policy* which minimizes the function, **R(p,q)**, called the *optimal* policy [32]. Thus the perception-action control posed in this work will hinge upon the determination of a set of optimal policies of the total return obtained starting in state p using an optimal policy, such that:

(2) $$f(p)=\min_{q}\left\{R(p,q)+\min f\left[T(p,q)\right]\right\}.$$

The equation (2) is the application of the “Principle of Optimality” of dynamic programming to the Markov decision process. The reciprocal screw system of the fifth order (5 DOFs) shall constitute complex lines on which wrenches act upon the body. These are triply infinite, or ∞^3^ lines, which satisfy one given condition, such that one solution to equation (1) exists between a screw and the reciprocal screw system, i.e., a manifold of constraint forces satisfying equation (1). It also follows that the reactions of the constraints by which the movements of the knee are confined to twist about a screw system of one DOF, can only be wrenches on the reciprocal screw system with an order of five (5 DOFs). The fact that each twist determines the wrench is the key to the method we now discuss. In the theory of perception-action control, we define approximation in policy space. A descriptive equation of the type appearing in equation (2) determines two functions, the return function f (p), and the policy function q (p). As such, the reactions of the constraints are only manifested by the success with which they resist the efforts of certain wrenches, i.e., the GRF, to disturb the equilibrium of the knee. Carrying out a control process in terms of following a policy is thus ideally made for dealing with chance or random effects. In the face of uncertainty, a natural response is to modify our behavior according to the actual run of events.

**Figure 2:**
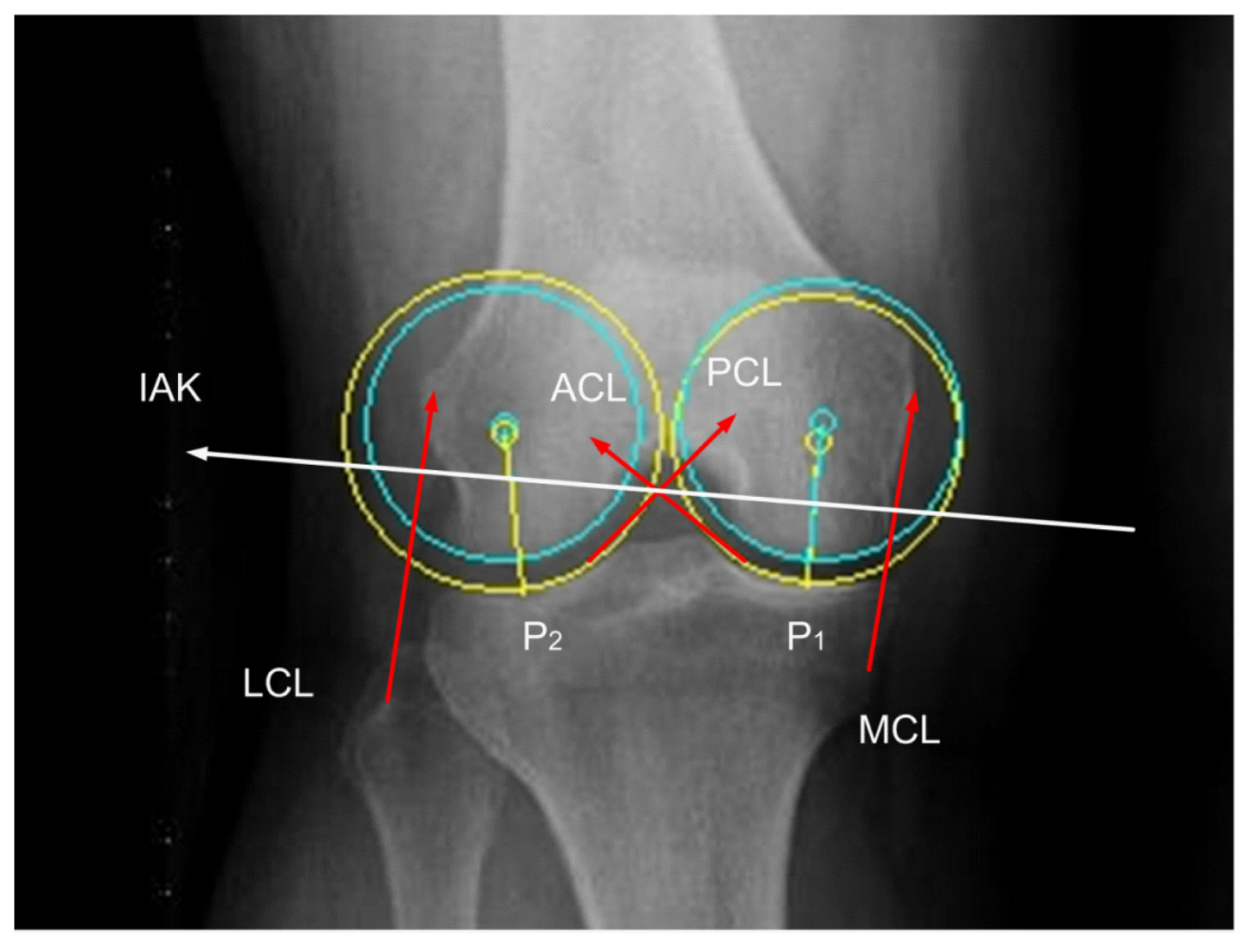
As previously described (Kim and Kohles, Kim et al.,) [22,29], the forces constraining the movement of the knee joint toward infinitesimal displacement are interlinked in terms of reciprocal conditions. The instantaneous axes of the knee (IAK) are reciprocal to five anatomic constraints: the cruciate ligament (ACL), the posterior cruciate ligament (PCL), the medial collateral ligament (MCL), the lateral collateral ligament (LCL), and the articular contact forces in both the medial (P1) and lateral (P2) compartments of the knee. P1 and P2 were taken as the common normal vectors to both curvatures as indicated. The reaction of the five constraints, which limit the motion of the knee, will neutralize every wrench on a screw within the constraint manifold that is reciprocal to the IAK. Thus, no work will be required to affect the movement along the IAK against forces at the constraints, which are reciprocal to the IAK.

Plücker [33,34] showed that if any screw motion about a certain axis be given to the acting force lines forming a linear complex, then these force lines still remain within the complex. Hence, if all lines of a complex are subjected to a screw motion about the axis, then the complex itself is not altered. We thus see that if a knee having freedom of the first order (1 DOF) is in equilibrium, then the forces that act upon the body shall reside within these line complexes in a screw system of the fifth order (5 DOFs). This system is in-turn reciprocal to the screw which defines the degree of freedom, without ceasing to belong to the overall complex (Figure 2).

As a further comparison, let us devise two ratios on this special configuration of a screw system of the first order of freedom (1 DOF):

(ratio)_1_=sum of the absolute values of the actuator twist-amplitudes divided by the amplitude of the end-effector twist, i.e., the foot=0
(ratio)_2_=sum of the absolute values of the actuator wrench-intensities divided by the intensity of the end-effector wrench=∞

Even though a loss of constraint about the IAK during knee motion, as indicated by (ratio)_2_, causes such static instability that a special configuration should be strictly avoided whenever the knee is being controlled during equilibrium, there are other conditions during locomotion where directional compliance is sought. In such a situation, a control mechanism of the IAK might create knee motion that replicates a special configuration at which the screw system that was applied *transitorily* is made to match the desired compliance [35].

### Ball-Disteli diagram

The Arnold-Kennedy theorem of three axes [36] may be manifested when two screws, **p_1_** and **p_2_**, result in a third screw, **p_IAK_** on cylindroids. In this application, consider the two screws as, **p_1_**, the twist of the shank, and, **p_2_**, the twist of the thigh, connected through the knee axis of the series. Since **p_1_** and **p_2_** are appropriated to two different elements of the mass-linkage, no kinematic significance can be attached to the composition of the two twists on **p_1_** and **p** If, however, the two twists on **p_1_** and **p_2_**, having the proper ratio of amplitudes, had been applied to a single rigid body, the displacement produced is one that could have been affected by a single twist about a single screw, IAK, on the cylindroids (**p_1_**,**p_2_**). If this intermediate screw is given, the ratio of the amplitude of the twists on the given screw may be determined as:

(3) $${\mathrm{dv}}_{1}p_{1}+{\mathrm{dv}}_{2}p_{2}+{\mathrm{dv}}_{\mathrm{IAK}}p_{\mathrm{IAK}}=0$$

where **dv_1_p_1_** is the twist of the shank, **dv_2_p_2_** is the twist of the thigh, and **dv_IAK_p_IAK_** is the relative displacement of the thigh with respect to the shank. The IAK is then defined by a linear combination of the two screws, **p_1_** and **p_2_** [36]. We can call upon the Ball-Disteli diagram that encapsulates the relation between the velocities of a pair of screws as well as the position and pitch of their relative screw motion, by referring to Plücker’s conoid, also known as Ball’s cylindroid [37,38].

**Figure 3:**
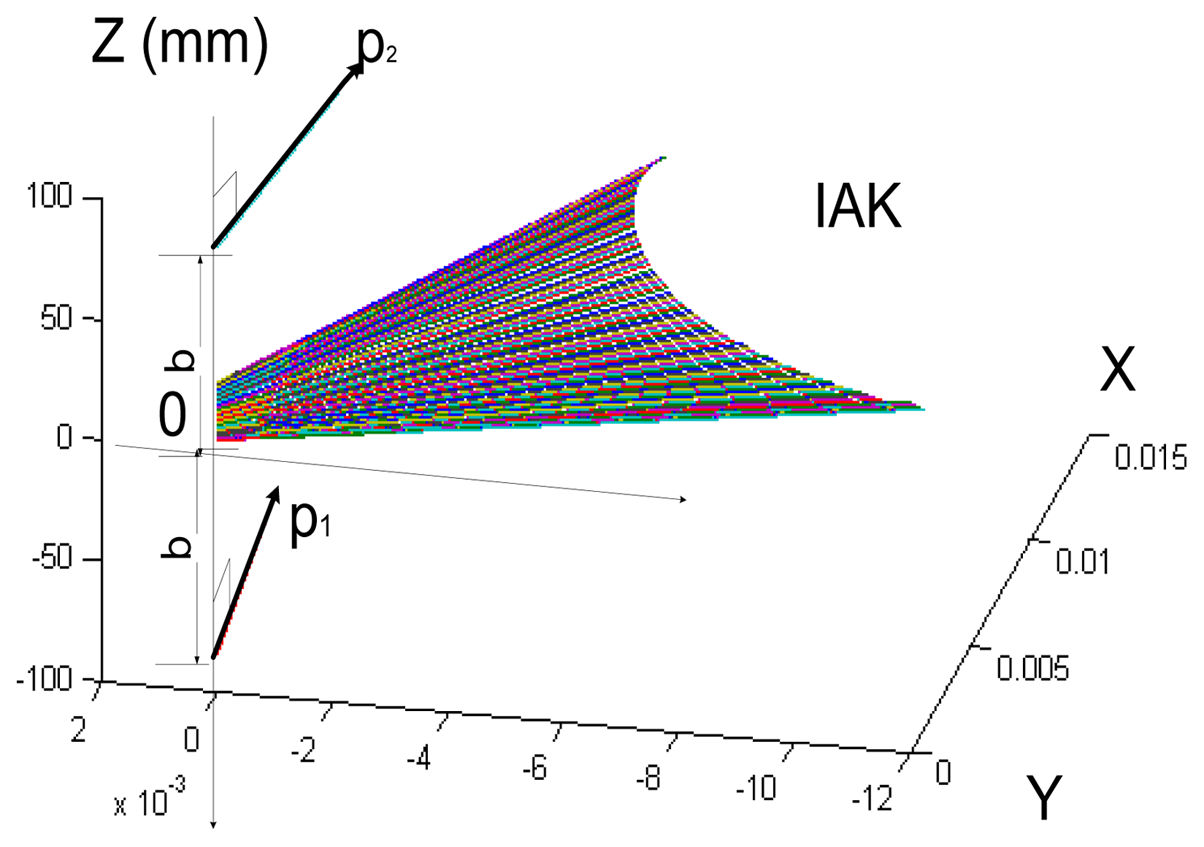
A functional schematic of the defined cylindroids, a cubic ruled surface the generators of which are the axes of the screws of a generalized two-screw system. We have described the general form of the one-parameter, two-screw system of linearly dependent screws, and have chosen a range of velocity ratios (0.3 to 2.5) as the physiologically feasible range, representing thigh versus shank function. Here, the solution for equation (1), which determines a unique IAK (p) to perceive and guide a mobility decision, is called the *policy* function.

In order to create the Ball-Disteli diagram, we start by taking two generally disposed screws, **p_1_** and **p_2_,** and for convenience place the z-axis along their common perpendicular with the half distance *b*. The origin of coordinate **o** is halfway between the screws and the x-axis is equally inclined by the angle, σ, to the screws (Figure 3). A screw for the IAK, that is linearly dependent on **p_1_** and **p_2_**, can be expressed as such after normalization:

(4) $$p_{\mathrm{IAK}}=\lambda_{\alpha }p_{\alpha }+\lambda_{\beta }p_{\beta }$$

where the corresponding coordinates of two screws on the principal screw can now be written as:

(5) $$p_{\alpha }\equiv \left[1,0,0;(b+\tan\sigma ),0,0\right]$$

(6) $$p_{\beta }\equiv \left[0,1,0;0,(-\frac{b}{\tan\sigma }),0\right]$$

Because **p***_α_* and **p***_β_* are centrally placed in the system and enjoy other special properties, these have been called the principal screws of the system [39]. Thereby, for a variable transmission ratio, $\frac{\lambda_{\alpha }}{\lambda_{\beta }}$, all relative axes are located on Plűcker’s conoid, which as revealed follows a cubic surface (Figure 3).

### Perception-Action coupling manifold

The screw system is designed ingeniously so that any deviation from equilibrium automatically generates a correcting force that tends to restore the system to equilibrium. In the simplest case, this restoring force is directly proportional to the disturbing forces. Although this case may appear to be the simplest, from the perception-action control point of view, control by policy is still more desirable. The perception-action coupling manifold, which is generated in terms of dual information surfaces during the stance phase of gait, is based on natural phenomena rather than an intellectual construct. In other words, perception-action coupling is a purely geometric representation of the subject-ground interaction. The perception-action coupling manifold, which utilizes correlating alignments of the mechanisms that generate the behavior of IAK and GRF through sensory feedback, can be used to investigate how a subject perceives affordances for effective locomotion [14]. The Ball-Disteli diagram will shift in position as the IAK shifts position so as to occupy a series of consecutive graphical positions in the diagram.

**Figure 4:**
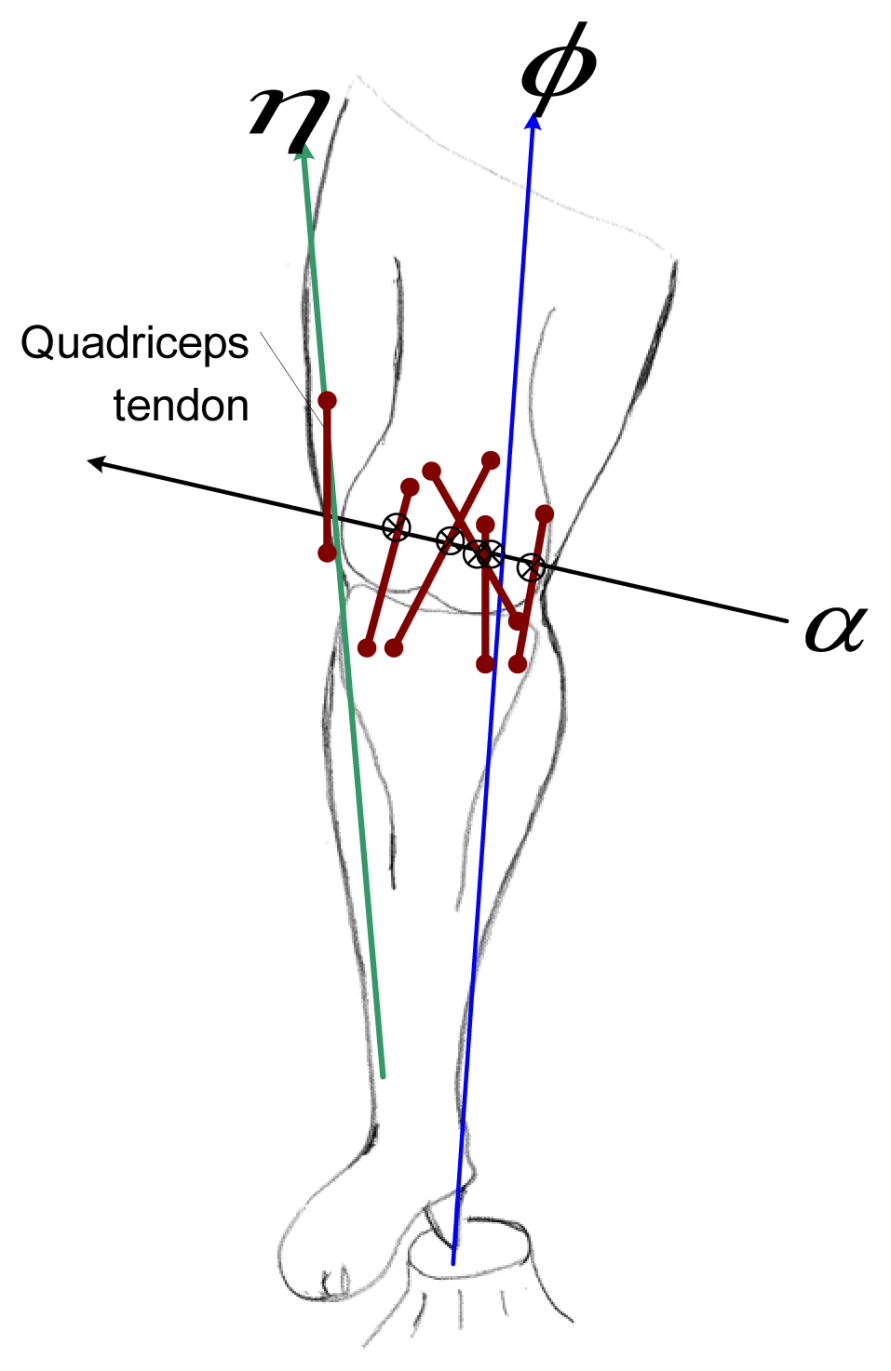
A schematic demonstrating the condition of joint equilibrium when a single wrench replaces two applied wrenches (GRF, *φ*, and muscle forces, *η*) such that one screw of the cylindroid representation is reciprocal to the IAK. Thus an impulsive wrench applied to the joint then makes the given screw, *α*, the instantaneous screw for the IAK. The joint reaction forces, *λ*, and force-couple or torque, *μ*, will be carried by the tissue structures shown as truncated lines. Other than the quadriceps tendon associated the muscle force vector, tendonous connections are not included in the constraint manifold due to their ability to function as elastic springs.

We can also determine the joint reactions due to the GRF in the same manner. If two wrenches act upon the knee, then the condition of equilibrium is met when the two wrenches are compounded by the aid of a cylindroid. For this condition, the single wrench that replaces the two wrenches shall lie upon that one screw of the cylindroid which is reciprocal to the IAK. The component wrenches within the reciprocal system are neutralized by the reactions of the constraints (Figure 4), while the remainder must compound into a wrench on a screw belonging to the screw system, which defines the freedom of the knee. We also perceived that a given wrench, GRF, *φ*, may be always replaced by a wrench of appropriate intensity on any other screw of muscles, in so far as the effect on a body only free to twist about is concerned. As such, we obtain:

(7) $$\eta^{\prime \prime }\delta_{\eta \$}+\varphi^{\prime \prime }\delta_{\varphi \$}=0.$$

Further, a wrench, can be always be expressed by a constraint force, at any point that resides on the IAK and a couple, in a plane through that point but not of course in general normal to the force (Figure 4). This statement should be clear since all the screws on the cylindroids are parallel to a plane. An analogy can also be made from the above equation to the simple problem of the condition where two forces should be unable to disturb the equilibrium of a particle, only free to move along a straight line.

The algorithm for calculating reciprocal screws using the nullspace operation was previously developed [40] and later translated into computation functions (MATLAB, Mathworks, Inc., Natick, MA) [41]. The algorithm has also been used to describe the reciprocal connections of the IAK as associated with knee joint constraints. Our study here used the described framework to predict the constraint reactions within a reciprocally connected knee joint model.

To validate our modeling approach for the IAK during the stance phase of gait, we used previously published experimental data sets and compared their measured medial and lateral contact forces with our predicted ones. Data included motion capture kinematics (x-y-z trajectories of markers located at the patella, shank, and thigh), fluoroscopy information, ground reaction forces, electromyographical data, medial and lateral knee contact forces, and strength data [42]. An available knee radiograph contained a view of the joint in the frontal plane and provided geometrical information regarding the constraint force vectors. Published data were collected from an instrumented right knee replacement implanted in an adult male subject (subject code JW, mass 65 kg, height 1.7 m). The gait trial for the subject demonstrated a medial-lateral trunk sway gait pattern similar to that reported previously [43].

## Results

**Figure 5:**
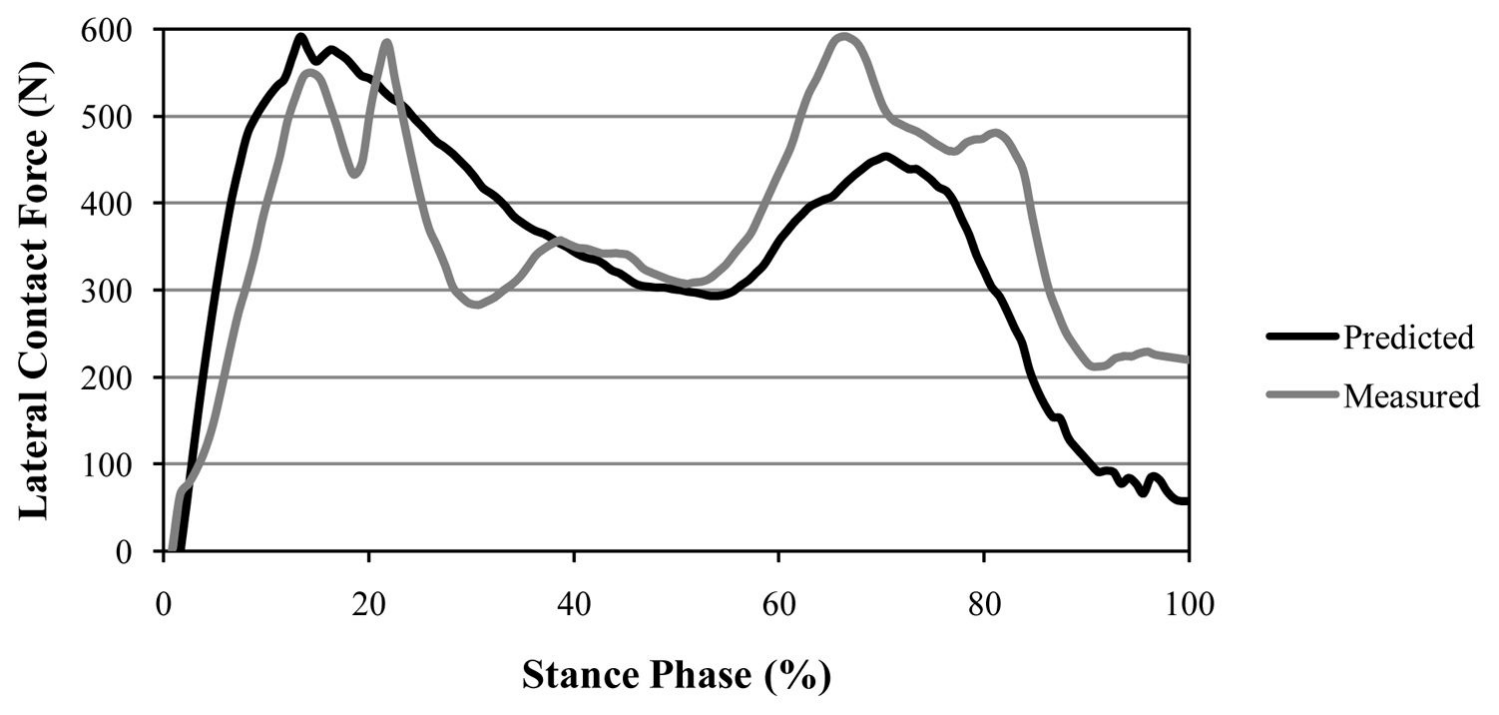
Comparison between the lateral force results of our theoretical approach and data gathered from an instrumented knee during trunk-sway gait trials (Fregly et al.) [42]. Differences between the predicted and measured values indicated an RMS error of 148.1 N.

**Figure 6:**
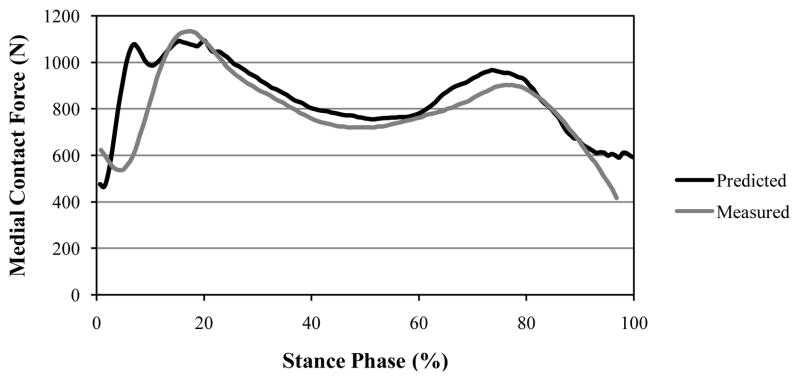
Comparison between the medial force results of the theoretical approach and data gathered from an instrumented knee (Fregly et al.) [42]. Again, differences between the predicted and measured values had an RMS error of 147.3 N.

The dynamic position of the IAK (Figure 3) was determined by the linear combination of two ISA’s of the shank and thigh (equation 4 in Section 2). If, however, the two twists, **p_1_** and **p_2_**, having the proper ratio of amplitudes, had been applied to a single rigid body, then the displacement produced could have been replaced by a single twist about a single screw IAK on the cylindroids (**p_1_,p_2_**). In the model presented in this paper, the one DOF equilibrium condition was considered such that the modeled knee was only free to twist about the IAK. A given GRF was replaced by a wrench of appropriate intensity on any muscle and both were compounded into a wrench, which is reciprocal to the IAK and resolved into component wrenches representing the lateral (Figure 5) and medial (Figure 6) contact forces belonging to the reciprocal screw system.

The measured instrumented knee data for the trunk-sway gait trials for the data sets were compared to the model prediction. The predicted maximum lateral and medial contact forces were 595.0 N and 1,092.0 N, respectively (Figures 5 and 6). The root mean square (RMS) errors during the contact of the foot with the ground were determined to be 148.1 N and 147.3 N for the lateral and medial forces, respectively (based on the stance phase shown in figures 5 and 6).

## Discussion

The purpose of this study was to present a general theory as a perception-to-action control algorithm characterizing knee joint motion. We proposed the perception-action coupling manifold as a mathematical means to connect the instantaneous screw of the knee with the ground reaction force as a modeling approach that was verified by defined tasks in an estimation of constraint reactions. The coupling manifold can be directly implemented in the mathematical sense of a ‘minimum information framework’. We have demonstrated that a perception-action coupling manifold, generated in terms of two screw axes surfaces during the stance phase, can explain the interaction between gait kinematics and external applied forces. Therefore, the shape of the reciprocal condition in the perception-action coupling manifold can be regarded as a ‘minimal unit of analysis’ of gait pattern under all load-bearing physiological conditions. As reported here, there exists a unique one-to-one correspondence between the GRF and the IAK. The unique characteristic of the GRF-IAK relationship provides a new perspective on this topic of investigation. This approach may provide a metric toward assessing clinical implications addressing gait disorders on an individual patient-specific basis.

We have demonstrated that locomotion is controlled by affordances perceived by the exemplar subject. Specifically, a given GRF wrench on the surface can be replaced by a muscle wrench of appropriate intensity with any other screw. Hence, a one degree of freedom constraint during locomotion was not inhibited by disturbances during the support phase within the environment, i.e., the stance phase. It also follows that the reactions of the constraints, by which the movements of a knee are confined to twist about the screws of a system IAK, can only be wrenches on the reciprocal screw system of the fifth order (5 DOFs). As such, the reactions of the constraints are only manifested by the success with which they resist the efforts of certain wrenches, i.e., the GRF, to disturb the equilibrium of the knee. We replaced these contact forces by a perception-action coupling manifold as operationalized parameters. We introduced the basic conceptual move to create and control manifolds out of carefully chosen (theoretically motivated) state variables [44].

In conclusion, the presented approach can measure ‘dynamic alignment’ of *in vivo* knee loads. One can align the IAK associated with the GRF to reduce the payload on the medial/lateral compartment, thus instead transmitting the reaction (braking) torque to the structure of the whole body. The reciprocal configuration (Figure 4) aligns a GRF with a reciprocal screw such that the reaction (braking) torques and forces on the joint are eliminated. Special orthotic or orthopedic treatments, which are rich in reciprocal configuration potential, may be designed for the post-treatment outcome of gait-related disorders. The optimal treatment incorporating the presented perspective requires further investigation. Overall, the essential geometric character of the described method seems particularly well adapted to provide an ecological solution for individual variability. Thus the mathematical framework articulates a sound foundation toward making gait analysis more diagnostically accurate.

Human biology is a field where complexities are raised in even the simplest models. The approach described here addresses the interface between psychology and physiology. In order to mathematically analyze physiologic control processes, we have introduced the basic concept of proprioceptive ‘information’, a term that does not refer to the highly specialized theory of coding [45]. In this application, we are thinking in a broader and more meaningful sense whereas our approach is based on the perception of affordances, i.e, possibilities for actions [14]. We have presented an approach, which has been neglected in contemporary modeling, toward examining the characteristics and accuracy of the environmental information available to the ambulating decision maker.
